# Natural history of recurrences in endometrial carcinoma

**DOI:** 10.3892/ol.2014.2362

**Published:** 2014-07-18

**Authors:** BENGT SORBE, CHRISTIAN JURESTA, CECILIA AHLIN

**Affiliations:** 1 Department of Oncology, University Hospital, Örebro S-70185, Sweden; 2 Department of Obstetrics and Gynecology, University Hospital, Örebro S-70185, Sweden

**Keywords:** endometrial carcinoma, recurrences, natural history, radiotherapy, chemotherapy

## Abstract

The aim of the present study was to evaluate the natural history of endometrial cancer recurrences with regard to predictive and prognostic factors. Between 1990 and 1999, 100 patients were treated for recurrences of endometrial carcinoma (all FIGO stages). Overall, 90 tumors were of endometrioid type. A total of 82 patients were treated with surgery, 41 patients received adjuvant external irradiation and 91 patients received vaginal brachytherapy. The median time to recurrence (TTR) was 32 months. The recurrences were treated using a combination of high-dose-rate brachytherapy and external pelvic irradiation in 35 cases. In addition, 44 patients were treated with chemotherapy and 21 patients received other types of therapy. The complete remission rate was 29% and the overall response rate was 44%. Among patients treated with radiotherapy, the response rate was 88% and, for those treated with chemotherapy, the rate was 33%. The local control of vaginal recurrences treated with combined radiotherapy was 93%. In 45 patients (45%) a second recurrence was identified and a third recurrence occurred in 12 patients. The overall five-year survival rate was 44%. Age, FIGO grade, nuclear grade, TTR and response to treatment were found to be independent and significant prognostic factors for overall survival rate. Locoregional recurrences were associated with a generalized extra-pelvic disease in 63% of the cases.

## Introduction

Endometrial cancer is the most common gynecological malignancy in western countries (1). The majority of endometrial carcinomas (ECs) are diagnosed at an early stage (FIGO I–II), exhibit a favorable prognosis and may be cured predominantly by primary surgery and adjuvant radiotherapy. However, 10–15% of tumors recur and the majority (80–90%) of recurrences take place within three years (2). Treatment of recurrences is a challenge. Local vaginal recurrences are curable if diagnosed early, however, pelvic and distant recurrences exhibit a poor prognosis. A number of predictive and prognostic factors have been studied previously, for example, age of the patient, time between treatment and recurrence (3), prior radiotherapy (4), histological type, FIGO grade, size of the tumor (5), type of therapy for the recurrent disease (6) and radiation dose to the target (7). However, the importance of these factors remains controversial. Notably, in randomized phase III trials, postoperative adjuvant radiotherapy significantly improved locoregional (vaginal and pelvic) tumor control, but did not improve survival rate (4,8). An explanation for this paradox may be found by investigating the natural history of the recurrences and the association between local, regional and distant sites of recurrences versus adjuvant therapy.

In the present retrospective study of 100 endometrial cancer recurrences, a number of predictive and prognostic factors were analyzed. The natural history of the recurrences was the predominant focus of the study and the importance of local, regional and distant recurrences for tumor control and survival were also analyzed and considered with regard to the various types of therapy administered and the outcome for these patients.

## Patients and methods

### Patients

Between January 1990 and December 1999 100 patients with recurrent EC were consecutively registered and treated at University Hospital (Örebro, Sweden). A total of 82 patients were treated primarily with abdominal hysterectomy and bilateral salpingo-oophorectomy, five patients with external radiotherapy and brachytherapy, 13 with brachytherapy alone, and two patients with chemotherapy. In 15 patients, lymph node sampling was performed and in four patients lymphadenectomy was performed.

A total of 30 patients exhibited vaginal recurrences and, in 21 patients, the first was a solitary vaginal recurrence. In nine cases the vaginal recurrence was combined with a pelvic recurrence or distant metastases. Furthermore, in 70 patients, the recurrence had extra-vaginal or multiple locations. Radiotherapy was the treatment selected for 35 patients predominantly using a combination of external beam therapy and vaginal brachytherapy. A total of 49 patients received chemotherapy (five in combination with radiotherapy). In 12 patients, local surgery in combination with chemotherapy or hormonal therapy was administered, three patients received surgery alone and 13 patients did not receive any treatment.

The study was approved by the Ethics Committee of Uppsala, Sweden (Dnr 2013/136).

### External radiotherapy

External beam pelvic irradiation was administered to 41 patients and vaginal brachytherapy to 91 patients. The total external dose varied between 40.0 and 46.0 Gy (mean dose, 44.4 Gy). The dose per fraction was 2.0 Gy with five fractions administered per week, whereby a four-field box-technique was used. The external treatment equipment used was 18-MV linear accelerators (Clinac IX, Varian Medical Systems, Inc., Palo Alto, CA, USA).

### Brachytherapy

A total of 13 patients were treated with intrauterine irradiation as the definitive primary therapy, and five patients with brachytherapy and external beam pelvic irradiation. The total brachytherapy dose varied between 5.0 and 48.0 Gy (mean dose, 23.8 Gy). The dose per fraction varied between 2.5 and 6.0 Gy. Brachytherapy was administered using the high dose-rate technique (MicroSelectron, Ir-192; Elekta Instrument AB, Stockholm, Sweden). The intrauterine treatment was administered using twin-applicators, and the vaginal treatment was administered using plastic cylinders (20, 25 or 30 mm in diameter) whereby the dose was specified 5 mm from the surface of the applicator. The upper two-thirds of the vaginal walls were defined as the target for adjuvant treatment and the whole vagina for the treatment of recurrent disease.

### Chemotherapy

A total of 49 patients received chemotherapy as the only treatment (n=35) or in combination with radiotherapy (n=5), hormonal therapy (n=4) or surgery (n=5). The most common chemotherapy regimen used (n=29) was a combination of teniposide, vincristine and cisplatin (VOP-regimen) (9). The second most common regimen (n=10) used was carboplatin and paclitaxel. Cisplatin-cyclophosphamide and cisplatin-doxorubicin were administered to two patients each, single drug carboplatin was administered to two patients and single drug cyclophosphamide was administered to four patients. The median number of courses administered first-line was seven (range, 1–13 courses). The number of regimens used varied between one and four. The median duration of chemotherapy treatment was 9 months (range, 1–49 months).

### Follow-up

All patients were treated and followed-up at University Hospital (Örebro, Sweden). During the first year following treatment the patients attended regular hospital visits every three months, then every four months during the second and third years, every six months up to five years and, following that, annually for up to ten years. The median follow-up time for surviving patients was 140 months (range, 51–185 months).

### Statistical analysis

For comparison of proportions, Pearson’s χ^2^ test was used and, for continuous variables, Student’s t-test was used for comparing independent groups. For variables with dichotomous outcomes the logistic regression analysis was used with univariate and multivariate techniques. The Kaplan-Meier method was used for the survival analyses. Cox proportional regression analysis was used to analyze prognostic factors with overall or cancer-specific survival rate as the endpoint. P<0.05 was considered to indicate a statistically significant difference. Statistica software, version 10 (StatSoft, Inc., Tulsa, OK, USA) was used for statistical analyses.

## Results

### Original stage distribution and histology of the tumors

The median age of the 100 patients with recurrent ECs was 70 years (range, 38–92 years). The original FIGO stage distribution is shown in Table I. A total of 85 tumors (85.0%) were stage I, eight tumors were stage II, six tumors were stage III and one tumor was stage IVB. Histological tumor type was identified as endometrioid in 90 cases (90.0%), serous carcinoma in eight cases, with one clear cell carcinoma and one undifferentiated carcinoma. Overall, 24 carcinomas were well differentiated, 54 moderately well-differentiated and 22 poorly differentiated. In 36 cases, the tumor infiltrated <50% of the myometrial thickness and, in 29 cases, the tumor infiltrated >50% (deep infiltration). In 58 tumors, the DNA ploidy was analyzed and 11 tumors (19.0%) were identified to be aneuploid. Lymphovascular space invasion was not regularly reported in this study of ECs. Overall, 29 carcinomas (29.0%) were classified as low-risk, 51 carcinomas (51.0%) as intermediate-risk and 20 carcinomas (20.0%) as high-risk.

### Primary therapy of the tumors

A total of 82 patients (82.0%) underwent primary surgery with total abdominal hysterectomy and bilateral salpingo-oophorectomy. Lymph node sampling was performed in 15 patients and pelvic lymphadenectomy in four patients. In 81 patients, no surgery was performed on the lymph nodes. A total of 41 patients (41.0%) received radiotherapy as primary radiotherapy or adjuvant postoperative pelvic irradiation, and 91 patients (91.0%) received vaginal brachytherapy as a primary therapy or postoperative adjuvant therapy. Furthermore, 11 patients received adjuvant chemotherapy due to high-risk factors. With the exception of three, all patients achieved primary cure (97.0%) of their ECs.

### Pattern of recurrences

A single vaginal recurrence was diagnosed as the first recurrence in 21 cases (21.0%). A vaginal and pelvic recurrence was diagnosed in two cases; in one case a combined vaginal and abdominal recurrence occurred, and in one case the sites of relapse included the vagina, pelvis and lungs. In an additional five cases, the vaginal recurrences were combined with multiple distant recurrences to the lymph nodes, liver and lungs. Overall, 30 vaginal recurrences were identified and, of these, 21 recurrences were solitary vaginal relapses. Pelvic recurrences were recorded in 16 cases, abdominal recurrences in 24 cases and distant recurrences in 59 cases. The median time period from diagnosis to the first recurrence was 32 months (range, 5–217 months).

In 45 patients (45.0%), a second recurrence was diagnosed, whereby six cases were identified as a novel vaginal recurrence and, in 39 cases, recurrence occurred at regional or distant sites, including the pelvis, abdomen, lymph nodes, liver, lungs, bone and central nervous system. The median time period between the first and second recurrence was 12 months (range, 1–146 months). In 12 patients (12.0%), a third relapse was recorded, four at the vaginal site and eight at various distant sites. The median time period between the second and third recurrence was 13 months (range, 3–70 months). Four cases experienced a forth relapse (all distant) and one patient exhibited a fifth relapse with metastasis to the pelvis and liver.

### Response rates

The overall response rate of the patients was 28.6% (22/77 patients). In the group treated with radiotherapy, the response rate was 87.5% (21/24 patients) and, in the chemotherapy group, the response rate was 33.3% (12/36 patients). In the group receiving other types of therapy, such as surgery or hormonal treatment, the response rate was 5.9%.

**Figure 1:**
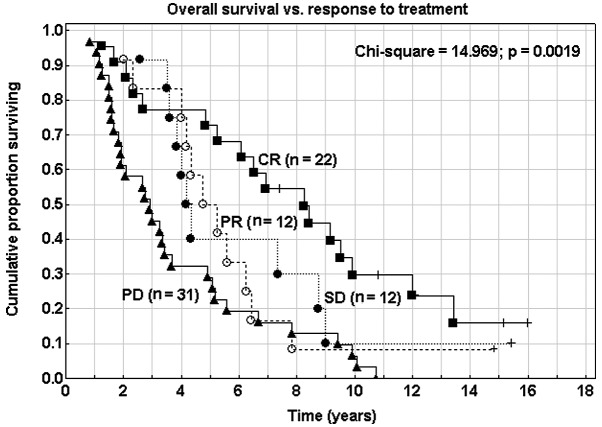
Comparison of overall survival rate with response to treatment. Overall survival rate was significantly higher in the patient group achieving CR compared with those not achieving CR (P=0.0019). No significant difference was identified between the PR and SD groups. Patients with PD exhibited a poorer prognosis. CR, complete response; PR, partial response; SD, stable disease; PD, progressive disease.

Response to administered therapy was significantly associated with the overall survival rate (P=0.002; Fig. 1). Patients achieving complete remission (CR) exhibited a 72.7% five-year survival rate whereas patients who did not achieve CR exhibited a survival rate of 35.7%.

### Local tumor control

The five-year local (vaginal) control rate was 88.3%. A total of 12 novel vaginal recurrences occurred during the follow-up period. Thus, 40.0% (12/30) vaginal recurrences recurred once. For the treatment of the first vaginal recurrence, 15 out of 21 patients received radiotherapy, external beam irradiation and vaginal brachytherapy in combination, and 14 of these patients (93.3%) achieved local control of the vaginal tumor. In six patients with vaginal recurrences, additional treatment types, such as surgery and chemotherapy, were administered; however only two of these patients (33.3%) achieved complete local control (P<0.001).

In the group of 15 patients achieving complete remission, 10 novel recurrences were identified as a second recurrence and, in three cases (20.0%), a novel vaginal recurrence was diagnosed. In seven cases, distant recurrences to the liver, lungs, lymph nodes and bone were identified. During the follow-up, an additional three recurrences were identified as a third recurrence (vagina plus pelvis and lung) and as a forth recurrence (vagina plus abdomen).

### Survival data of the patients

At the time of follow-up 10 patients (10.0%) had survived, 77 patients had succumbed to the disease, and 13 patients had succumbed to intercurrent diseases. The median follow-up time for surviving patients was 140 months (range, 51–185 months).

**Figure 2:**
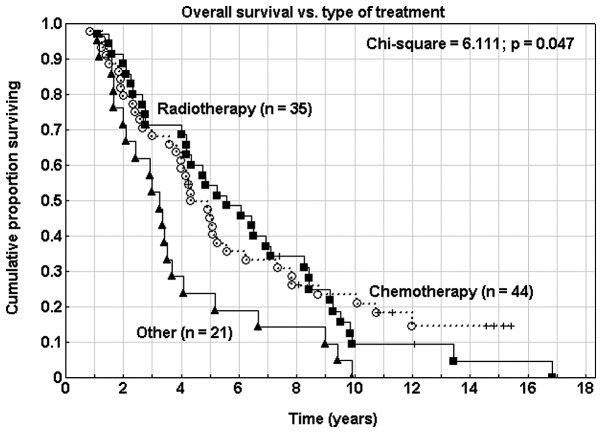
Comparison of overall survival rate with main treatment type (radiotherapy, chemotherapy or other therapy). Patients not treated with radiotherapy or chemotherapy (surgery alone, hormones alone or no active treatment) exhibited a decreased overall survival rate compared with that of patients in the remaining two groups (P=0.047).

The overall five-year survival rate of the patients was 43.9% [95% confidence interval (CI), 34.1–53.7%] and the cancer-specific survival rate was 47.0% (95% CI, 37.0–57.0%). Females exhibiting vaginal recurrences alone exhibited an increased overall survival rate (61.9%) when compared with females with recurrences at other sites (39.0% at five years); however, no significant differences were identified (P=0.286). Patients with FIGO stage III tumors exhibited a five-year overall survival rate of 35.3%. Overall survival rate was significantly associated with treatment type of the recurrent disease (P=0.047). Patients who did not receive radiotherapy or chemotherapy (n=21) exhibited a decreased overall survival rate (Fig. 2). Furthermore, no significant difference was identified between overall survival of patients treated with surgery alone (n=3), hormonal therapy alone (n=5) or with no active therapy (n=13) (P=0.425). The overall five-year survival rate of patients following the first recurrence was 19.2% (95% CI, 11.3–26.1%) and the median survival time was 14.8 months. In the group that did not receive radiotherapy or chemotherapy, the survival rate was 5.4% (P<0.001). No significant difference in survival rate was identified between patients treated with radiotherapy, chemotherapy or a combination of the two.

### Cox proportional regression analyses

In univariate Cox proportional regression analysis, patient age, time to recurrence (TTR), FIGO- and nuclear grade of the tumor, the risk group and response to therapy were significantly associated with overall survival rate. However, the original tumor stage, DNA ploidy, myometrial infiltration and lymph node surgery (sampling or lymphadenectomy) were not associated with the survival rate in this study (Table II). Patient age (P<0.001), time interval to recurrence (P<0.001), FIGO grade (P=0.048), nuclear grade of the tumor (P=0.001) and response to therapy (P<0.001) were also identified as significant prognostic factors in multivariate Cox analysis with regard to overall survival rate (Table III). The site of recurrence was not found to be significant with regard to overall survival rate.

No significant difference in overall survival rate was identified between patients that received external beam therapy as part of their primary treatment and patients that received surgery alone or surgery in addition to vaginal brachytherapy (P=0.322). Adjuvant vaginal brachytherapy was not associated with overall survival rate (P=0.927). In addition, the probability of achieving complete vaginal remission was higher (100.0 vs. 67.3%) in the group receiving no adjuvant brachytherapy as part of the first-line treatment; however, no significant differences were identified (P=0.269). Type of treatment for the recurrences in the complete series is presented in Table IV.

### Predictive and prognostic factors for treatment outcome

The primary risk group of the tumor was also significantly associated with the outcome of the treatment of the vaginal recurrence. A total of 73.4% of patients with tumors belonging to a low-risk group achieved complete remission, 42.1% in the medium risk group and 0.0% in the high-risk group.

In addition, TTR was significantly associated with overall survival rate (P=0.002). The risk of mortality decreased 2.1% per month with increasing TTR. This was also observed for cancer-specific survival rate (P=0.001) and local (vaginal) and locoregional (vaginal or pelvic) recurrences in separate analyses (P<0.001).

## Discussion

EC has a favorable prognosis, however, 10–15% patients relapse (10) and the five-year overall survival rate is 80% (11). It has become common to identify three risk groups (high, medium and low) among EC patients with different survival and recurrence rates, as well as different patterns of recurrence. Vaginal recurrences are the most frequent type of recurrences in all risk groups and also the type of relapse that is possible to cure if diagnosed early (8,12). The risk group distribution for high-, medium- and low-risk groups was 20, 51 and 29%, respectively. The endometrioid type constituted 90% of the cases and 22% were poorly differentiated. A total of 91 patients (91%) received postoperative adjuvant brachytherapy and 41 patients (41%) received external beam irradiation to the pelvic region.

In 21 patients, single vaginal recurrences were diagnosed and, in nine patients, vaginal relapse in combination with pelvic, abdominal, liver, lung or lymph node metastases was identified. The median time between diagnosis and recurrence was 32 months, which is consistent with previous studies (3). A second recurrence was recorded in 45 patients (45%), whereby six were vaginal and 39 were regional or distant. In 17 patients, ≥3 recurrences were recorded. The median time between the first and the second recurrence was 12 months.

In the present study, 76% (16/21 patients) vaginal recurrences achieved primary cure. Following a combination of brachytherapy and external beam therapy a cure rate of 93% was achieved. However, in the group receiving other therapies, only 33% achieved local control and this was significantly lower than that in the radiotherapy group. Hasbini *et al* reported the same local control rate in a study of 23 patients (13). Among 10 patients with a second, third or fourth vaginal recurrence, seven patients succumbed to the disease. The five-year cumulative local control rate was 67%. This was comparable to the results of Pai et al (14), whereby a 90% complete response rate and a 74% 10-year cumulative local control rate was reported in a series of 20 patients. Furthermore, Jhingran *et al* (6) reported a five-year local control rate of 69% in a study of 91 patients. In the PORTEC-1 trial, 35 isolated vaginal recurrences were treated with curative intent (external radiotherapy and brachytherapy, and in some cases surgery), and 89% achieved complete remission and 77% achieved long-term remission (4). In a study of 22 patients with isolated vaginal recurrences, Petignat *et al* (15) reported an 100% complete response rate whereby no patients exhibited locoregional recurrences. However, Hart *et al* (16) reported a 54% failure rate following radiotherapy in 26 tumor recurrences. Brachytherapy was not used as a routine treatment in these patients. External beam therapy with or without adjuvants is not sufficient to replace vaginal brachytherapy in the treatment of vaginal relapses. In the present study, nine cases of distant metastasis (30%) were identified with the vaginal recurrence as the first recurrence and during follow-up and, following treatment, an additional 15 regional or distant recurrences (50%) were identified. Therefore, 52% (11/21) isolated vaginal recurrences recurred distantly following locoregional treatment with radiotherapy (17).

The overall five-year survival rate of the patients was 44% and the cancer-specific survival was 47%. In a study by Wylie *et al* (18), of 58 recurrences, an overall survival rate of 53% and a local control rate of 65% were identified. Lin *et al* (5) also reported a rate of 53% in a study of 50 patients. Colombo *et al* (19) reported a 57% survival rate without evidence of disease between three and 11 years following treatment. By contrast, Blecharz *et al* (20) reported a five-year overall survival rate of only 42% in 47 patients that received treatment for vaginal recurrences and the corresponding rate for pelvic recurrences was 13%. A similar survival rate was reported by Jhingran *et al* (6), with 43% overall survival at five years. Creutzberg *et al* (4) identified a difference in survival following the treatment of vaginal relapses between two groups with or without prior external radiotherapy (PORTEC-1 study); the five-year survival rates were 43 and 65%, respectively. However, in the present study, such a difference in survival rate was not identified. TTR for all recurrences was a significant prognostic factor for cancer-specific and overall survival rate but not for patients with locoregional relapses. In addition, Robbins *et al* (3) reported that a TTR of <18 months was associated with shorter overall and cancer-specific survival, but only in patients with extra-pelvic recurrence.

In univariate Cox analyses, FIGO grade, nuclear grade, the original risk group of the tumor (low-, medium- or high-risk), TTR and response type, as well as patient age were statistically significant prognostic factors for overall survival rate. However, in multivariate analysis only age, FIGO grade, nuclear grade, TTR and type of response (complete remission) were found to be independent and significant prognostic factors for overall survival rate. This was also identified for cancer-specific survival rate. Furthermore, previous studies have identified additional predictive and prognostic factors. Lin *et al* (5) reported that age, FIGO grade and size of the recurrence were significant predictors of overall survival. In addition, Blecharz *et al* (20) found that recurrence site (vaginal versus pelvic recurrences) was the only independent prognostic factor for five-year overall survival. Hasbini *et al* (13) revealed that extra-vaginal extension, tumor size and stage of initial disease had a significant impact on the prognosis. Smaniotto *et al* (21) presented a scoring system (time between surgery and recurrence, pelvic wall site, positive lymph nodes, hemoglobin levels <11 g/dl) to identify patients benefiting from treatment. Patients with a low score (<2) exhibited a significantly improved outcome, local control of the disease and overall survival when compared with patients with a score of ≥2. Jhingran *et al* (6) found that external beam irradiation in addition to brachytherapy versus single modality therapy was significant in univariate analysis with regard to overall survival rate. In a multivariate study of 73 endometrial cancer recurrences, Jereczek *et al* (7) reported that only the stage of recurrent disease and a high total irradiation dose were found to correlate with improved survival.

Toxicity was not evaluated in this study. However, from previous studies using similar treatment techniques of vaginal recurrences 14% grade 3 toxicity of the vagina and 11% grade 3–4 gastrointestinal toxicity was recorded (22). Pai *et al* (14) reported a late complication rate of 15% and no grade 3 or 4 late complications. Petignat *et al* (15) reported 18% late grade 3–4 gastrointestinal toxicity and 50% grade 3 vaginal toxicity. Nag *et al* (23) reported 13 patients with interstitial brachytherapy for salvage of vaginal recurrence, whereby all tumors were locally controlled; however, long-term morbidity was 15%, including vaginal ulceration, colorectal fistula and grade 2 proctitis.

An important conclusion from the present study was that radiotherapy (high-dose rate vaginal brachytherapy and external beam irradiation) is an important part of the therapy to achieve local tumor control. However, survival is dependent on extra-vaginal tumor localization and, in this study, 79% of patients exhibited regional or distant metastases at diagnosis and 50% of local recurrences showed novel recurrences at distant sites during the follow-up period. With regard to locoregional recurrences, 63% were part of a generalized, extra-pelvic tumor spread at diagnosis or during follow-up. This may provide an explanation for the failure of locoregional adjuvant therapy for prolonging survival despite improved locoregional tumor control. This failure was also evident in the treatment of recurrent disease, as shown in the present study. Radiotherapy exhibited the highest locoregional control rate (71%); however, survival was similar to chemotherapy-treated patients with 11% complete clinical remission.

In conclusion, in the current study, 21% of patients exhibited single vaginal recurrences and 93% of cases were cured using combined radiotherapy (external beam irradiation in combination with vaginal brachytherapy). Thus, 79% of patients exhibited regional or distant metastases at the diagnosis of the first recurrence and 52% of the primary isolated vaginal recurrences recurred as distant metastases. Age, tumor grade, TTR and therapy responses were significant prognostic factors. Locoregional control was achieved by radiotherapy; however, survival was similar to that with chemotherapy alone and was significantly improved when compared with other types of therapy or no active treatment.

## Figures and Tables

**Table I:** Original FIGO stage distribution and histopathological characteristics of the tumors.

Parameter	n	%
FIGO stage		
IA	42	42.0
IB	39	39.0
IC	4	4.0
IIA	3	3.0
IIB	5	5.0
IIIA	4	4.0
IIIC	2	2.0
IVB	1	1.0
Histology		
Endometrioid	90	90.0
UPSC (serous)	8	8.0
Clear cell	1	1.0
Undifferentiated	1	1.0
FIGO grade		
Well differentiated	24	24.0
Moderately well-differentiated	54	54.0
Poorly differentiated	22	22.0
DNA ploidy		
Diploid	47	47.0
Aneuploid	11	11.0
Unknown	42	42.0
S-phase fraction (%)	7.3 (mean)	1.0–20.0 (range)
Myometrial invasion		
Superficial (<50%)	52	52.0
Deep (≥50%)	48	48.0
Risk groups		
Low	29	29.0
Medium	51	51.0
High	20	20.0

UPSC, uterine papillary serous carcinoma.

**Table II:** Univariate Cox proportional regression analyses. Prognostic factors of the primary tumor and the recurrence for overall survival rate.

Prognostic factor	β	Standard error	Risk ratio	95% confidence interval	P-value
Age (per year)	0.033	0.013	1.034	1.009–1.060	0.008
Primary tumor					
FIGO-stage (per stage)	0.021	0.163	1.021	0.742–1.406	0.898
FIGO-grade (3 vs. 1–2)	0.633	0.265	1.883	1.119–3.168	0.017
Nuclear grade (3 vs. 1–2)	1.088	0.353	2.968	1.487–5.925	0.002
DNA ploidy^a^	0.155	0.343	1.168	0.597–2.285	0.651
Infiltration^b^	0.218	0.247	1.244	0.767–2.018	0.376
Risk groups (3 vs. 1–2)	0.898	0.263	2.454	1.465–4.112	<0.001
Lymph node surgery^c^	0.311	0.258	1.364	0.823–2.261	0.229
External beam RT^c^	0.214	0.215	1.238	0.812–1.888	0.321
Vaginal RT^c^	−0.031	0.354	0.970	0.485–1.940	0.930
Adjuvant chemotherapy^c^	0.498	0.324	1.645	0.872–3.103	0.124
Recurrence					
Time to recurrence (months)	−0.013	0.004	0.988	0.980–0.995	0.002
Treatment (CT vs. RT)	−0.048	0.232	0.953	0.605–1.503	0.837
Treatment response^d^	1.595	0.323	4.927	2.618–9.273	<0.001
Site of recurrence^e^	0.208	0.247	1.232	0.760–1.996	0.398
Number of recurrences	−0.110	0.128	0.896	0.697–1.151	0.389

a Non-diploid vs. diploid;

b ≥50 vs. <50%;

c yes vs. no;

d non-CR vs. CR;

e distant vs. locoregional.

RT, radiotherapy; CT, chemotherapy; CR, complete remission.

**Table III:** Multivariate Cox proportional regression analysis. Prognostic factors of the primary tumor and the recurrence for overall survival rate.

Prognostic factor	β	Standard error	Risk ratio	95% confidence interval	P-value
Patient					
Age (per year)	0.046	0.013	1.047	1.021–1.074	<0.001
Primary tumor					
FIGO-grade^a^	0.544	0.275	1.723	1.004–2.956	0.048
Nuclear grade^a^	1.272	0.391	3.569	1.660–7.674	0.001
Recurrence					
Time to recurrence^c^	−0.022	0.005	0.978	0.969–0.988	<0.001
Treatment response^b^	1.595	0.323	4.927	2.618–9.273	<0.001

a Grade 3 vs. grade 1–2;

b non-CR vs. CR;

c risk decrease per month.

CR, complete remission.

**Table IV:** Treatment of the recurrences of the complete series (n=100).

Treatment	n	%
Surgery	12	12.0
± Radiotherapy or chemotherapy		
Radiotherapy	35	35.0
External beam therapy + vaginal brachytherapy		
Chemotherapy	49	49.0
± Surgery, radiotherapy or hormonal therapy		
Hormonal therapy	10	10.0
± Radiotherapy or chemotherapy		
No active treatment	13	13.0
